# 1,1′-Bis(4-fluoro­phen­yl)-3,3′-diisobutyl-4,4′-diphen­oxy-1*H*,1′*H*-4,4′-bipyrazole-5,5′(4*H*,4′*H*)-dione

**DOI:** 10.1107/S1600536811011664

**Published:** 2011-04-07

**Authors:** Hoong-Kun Fun, Madhukar Hemamalini, R. Venkat Ragavan, V. Vijayakumar, M. Venkatesh

**Affiliations:** a X-ray Crystallography Unit, School of Physics, Universiti Sains Malaysia, 11800 USM, Penang, Malaysia; b Organic Chemistry Division, School of Advanced Sciences, VIT University, Vellore 632 014, India

## Abstract

In the title compound, C_38_H_36_F_2_N_4_O_4_, the pyrazole rings form dihedral angles of 50.02 (4) and 18.05 (4)° with their attached fluorobenzene rings, and make dihedral angles of 76.08 (4) and 73.54 (5)° with the aromatic ring of the attached phen­oxy group. In the crystal, the molecules are connected by weak C—H⋯π inter­actions.

## Related literature

For the synthesis and applications of pyrazole derivatives, see: Venkat Ragavan *et al.* (2009 ▶, 2010 ▶). For the stability of the temperature controller used in the data collection, see: Cosier & Glazer (1986 ▶).
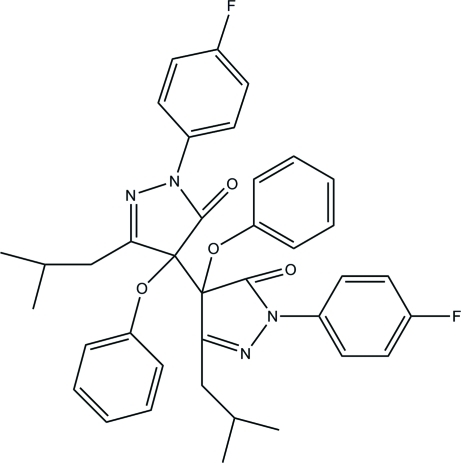


## Experimental

### 

#### Crystal data

C_38_H_36_F_2_N_4_O_4_
*M* *_r_* = 650.71
Monoclinic, 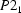

*a* = 11.3875 (5) Å
*b* = 11.4582 (5) Å
*c* = 13.4885 (6) Å
β = 109.752 (1)°
*V* = 1656.43 (13) Å^3^
*Z* = 2
Mo *K*α radiation
μ = 0.09 mm^−1^
*T* = 100 K
0.53 × 0.21 × 0.14 mm

#### Data collection

Bruker APEXII DUO CCD area-detector diffractometer
Absorption correction: multi-scan (*SADABS*; Bruker, 2009 ▶) *T*_min_ = 0.953, *T*_max_ = 0.987
26247 measured reflections
6956 independent reflections
6490 reflections with *I* > 2σ(*I*)
*R*_int_ = 0.033

#### Refinement

*R*[*F* ^2^ > 2σ(*F* ^2^)] = 0.036
*wR*(*F* ^2^) = 0.097
*S* = 1.03
6956 reflections
437 parameters
1 restraint
H-atom parameters constrained
Δρ_max_ = 0.38 e Å^−3^
Δρ_min_ = −0.19 e Å^−3^

### 

Data collection: *APEX2* (Bruker, 2009 ▶); cell refinement: *SAINT* (Bruker, 2009 ▶); data reduction: *SAINT*; program(s) used to solve structure: *SHELXTL* (Sheldrick, 2008 ▶); program(s) used to refine structure: *SHELXTL*; molecular graphics: *SHELXTL*; software used to prepare material for publication: *SHELXTL* and *PLATON* (Spek, 2009 ▶).

## Supplementary Material

Crystal structure: contains datablocks global, I. DOI: 10.1107/S1600536811011664/rz2574sup1.cif

Structure factors: contains datablocks I. DOI: 10.1107/S1600536811011664/rz2574Isup2.hkl

Additional supplementary materials:  crystallographic information; 3D view; checkCIF report

## Figures and Tables

**Table 1:** Hydrogen-bond geometry (Å, °) *Cg1* is the centroid of the N2=C8 double bond. *Cg3* and *Cg5* are the centroids of the C1–C6 and C19–C24 rings, respectively.

*D*—H⋯*A*	*D*—H	H⋯*A*	*D*⋯*A*	*D*—H⋯*A*
C15—H15*A*⋯*Cg*5^i^	0.93	2.82	3.6941 (14)	157
C20—H20*A*⋯*Cg*1	0.93	2.54	2.9632 (12)	135
C29—H29*A*⋯*Cg*3^ii^	0.93	2.82	3.4701 (15)	128
C36—H36*A*⋯*Cg*3^i^	0.98	2.91	3.7529 (15)	145

Symmetry codes: (i) 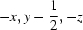; (ii) 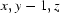.

**Fig. 1.**
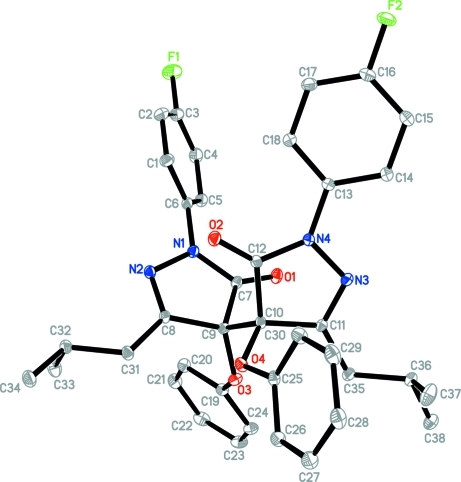
The molecular structure of the title compound, showing 30% probability displacement ellipsoids. Hydrogen atoms are omitted for clarity.

**Fig. 2.**
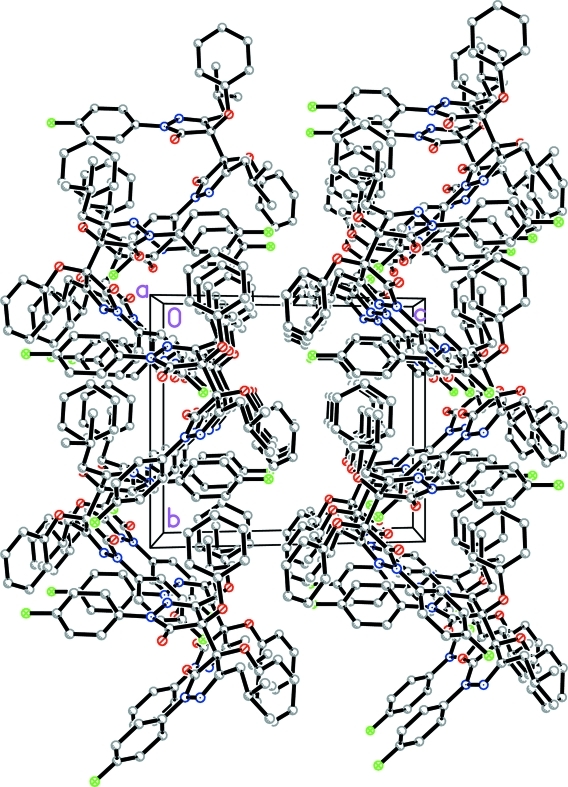
Crystal packing of the title compound viewed along the a axis. Hydrogen atoms are omitted for clairty.

## supplementary crystallographic information

### Comment

Antibacterial and antifungal activities of the azoles are most widely studied and some of them are in clinical practice as anti-microbial agents. However, the azole-resistant strains has led to the development of new antimicrobial compounds. In particular pyrazole derivatives are extensively studied and used as antimicrobial agents.  Pyrazole is an important class of heterocyclic compound and many pyrazole derivatives are reported to have the broad spectrum of biological activities, such as anti-inflammatory, antifungal, herbicidal, anti-tumour, cytotoxic, molecular modelling, and antiviral activities. Pyrazole derivatives also act as antiangiogenic agents, A3 adenosine receptor antagonists, neuropeptide YY5 receptor antagonists, kinase inhibitor for treatment of type 2 diabetes, hyperlipidemia, obesity and thrombopiotinmimetics. Recently urea derivatives of pyrazoles have been reported as potent inhibitors of p38 kinase. Since the high electronegativity of halogens (particularly chlorine and fluorine) in the aromatic part of the drug molecules play an important role in enhancing their biological activity, we are interested to have 4-fluoro or 4-chloro substitution in the aryls of 1,5-diaryl pyrazoles.  As part of our on-going research aiming the synthesis of new antimicrobial compounds, we have reported the synthesis of novel pyrazole derivatives and their microbial activities (Venkat Ragavan *et al.*, 2009, 2010).

In the molecule of the title compound (Fig. 1), the pyrazole rings (N1–N2/C7–C9; N3–N4/C10–C12) form dihedral angles of 50.02 (4) and 18.05 (4)° with the attached benzene rings (C1–C6; C13–C18), and of 76.08 (4) and 73.54 (5)° with the aromatic ring of the attached phenoxy group (C19–C24; C25–C30). In the crystal structure (Fig. 2), there is no classical hydrogen bond and stabilization is provided by weak C—H···π interactions (Table 1), involving the centroids of the N2═C8 double bond (centroid Cg1), C1–C6 ring (centroid Cg3) and C19–C24 ring (centroid Cg5).

### Experimental

1-(4-Fluorophenyl)-3-isobutyl-4-phenoxy-1*H*-pyrazole- 5(4*H*)-one was synthesized using the method reported in the literature (Venkat Ragavan *et al.*, 2010) and was converted into the title compound under the experimental condition. Single crystals of the title compound were obtained byu slow evaporation of an ethanol / chloroform (1:1 *v*/*v*) solution. Yield: 52%. M. p. 198 °C.

### Refinement

All H atoms were positioned geometrically and refined using a riding model, with C–H = 0.93–0.97 Å and with with *U*_iso_(H) = 1.2 *U*_eq_(C) or 1.5 *U*_eq_(C) for methyl H atoms.  A rotating group model was applied to the methyl groups. 4666 Friedel pairs were merged in the final refinement cycles.

### Crystal data

**Table:**

C_38_H_36_F_2_N_4_O_4_	*F*(000) = 684
*M**_r_* = 650.71	*D*_x_ = 1.305 Mg m^−^^3^
Monoclinic, *P*2_1_	Mo *K*α radiation, λ = 0.71073 Å
Hall symbol: P 2yb	Cell parameters from 9923 reflections
*a* = 11.3875 (5) Å	θ = 2.6–33.9°
*b* = 11.4582 (5) Å	µ = 0.09 mm^−^^1^
*c* = 13.4885 (6) Å	*T* = 100 K
β = 109.752 (1)°	Block, colourless
*V* = 1656.43 (13) Å^3^	0.53 × 0.21 × 0.14 mm
*Z* = 2	

### Data collection

**Table:**

Bruker APEXII DUO CCD area-detector diffractometer	6956 independent reflections
Radiation source: fine-focus sealed tube	6490 reflections with *I* > 2σ(*I*)
graphite	*R*_int_ = 0.033
φ and ω scans	θ_max_ = 34.0°, θ_min_ = 1.9°
Absorption correction: multi-scan (*SADABS*; Bruker, 2009)	*h* = −17→17
*T*_min_ = 0.953, *T*_max_ = 0.987	*k* = −16→17
26247 measured reflections	*l* = −21→21

### Refinement

**Table:**

Refinement on *F*^2^	Primary atom site location: structure-invariant direct methods
Least-squares matrix: full	Secondary atom site location: difference Fourier map
*R*[*F*^2^ > 2σ(*F*^2^)] = 0.036	Hydrogen site location: inferred from neighbouring sites
*wR*(*F*^2^) = 0.097	H-atom parameters constrained
*S* = 1.03	*w* = 1/[σ^2^(*F*_o_^2^) + (0.0646*P*)^2^ + 0.0777*P*] where *P* = (*F*_o_^2^ + 2*F*_c_^2^)/3
6956 reflections	(Δ/σ)_max_ < 0.001
437 parameters	Δρ_max_ = 0.38 e Å^−^^3^
1 restraint	Δρ_min_ = −0.19 e Å^−^^3^

### Special details

**Table:**

Experimental. The crystal was placed in the cold stream of an Oxford Cryosystems Cobra open-flow nitrogen cryostat (Cosier & Glazer, 1986) operating at 100.0 (1) K.
Geometry. All s.u.'s (except the s.u. in the dihedral angle between two l.s. planes) are estimated using the full covariance matrix. The cell s.u.'s are taken into account individually in the estimation of s.u.'s in distances, angles and torsion angles; correlations between s.u.'s in cell parameters are only used when they are defined by crystal symmetry. An approximate (isotropic) treatment of cell s.u.'s is used for estimating s.u.'s involving l.s. planes.
Refinement. Refinement of F^2^ against ALL reflections. The weighted R-factor wR and goodness of fit S are based on F^2^, conventional R-factors R are based on F, with F set to zero for negative F^2^. The threshold expression of F^2^ > 2σ(F^2^) is used only for calculating R-factors(gt) etc. and is not relevant to the choice of reflections for refinement. R-factors based on F^2^ are statistically about twice as large as those based on F, and R- factors based on ALL data will be even larger.

### Fractional atomic coordinates and isotropic or equivalent isotropic displacement parameters (Å^2^)

**Table:**

	*x*	*y*	*z*	*U*_iso_*/*U*_eq_	
F1	0.16157 (12)	0.88614 (12)	−0.18115 (9)	0.0445 (3)	
F2	0.06578 (9)	0.23698 (10)	−0.42001 (6)	0.02846 (19)	
O1	0.13281 (8)	0.47312 (9)	0.13477 (8)	0.01997 (17)	
O2	0.43391 (8)	0.32469 (9)	0.06204 (7)	0.01912 (17)	
O3	0.31599 (9)	0.37927 (8)	0.33952 (7)	0.01736 (16)	
O4	0.43323 (8)	0.21142 (8)	0.26514 (7)	0.01669 (15)	
N1	0.31858 (9)	0.55722 (10)	0.13450 (8)	0.01593 (17)	
N2	0.44800 (9)	0.54499 (10)	0.19062 (8)	0.01617 (17)	
N3	0.15255 (9)	0.20611 (10)	0.06656 (8)	0.01630 (17)	
N4	0.23161 (9)	0.25123 (10)	0.01456 (7)	0.01599 (17)	
C1	0.33645 (12)	0.64873 (14)	−0.02151 (10)	0.0223 (2)	
H1A	0.4009	0.5976	−0.0188	0.027*	
C2	0.29730 (14)	0.73220 (16)	−0.10091 (11)	0.0280 (3)	
H2A	0.3357	0.7384	−0.1516	0.034*	
C3	0.20009 (14)	0.80572 (14)	−0.10295 (11)	0.0273 (3)	
C4	0.14072 (13)	0.80168 (13)	−0.02921 (11)	0.0250 (2)	
H4A	0.0760	0.8527	−0.0327	0.030*	
C5	0.18107 (12)	0.71836 (12)	0.05087 (10)	0.0198 (2)	
H5A	0.1433	0.7135	0.1020	0.024*	
C6	0.27778 (10)	0.64274 (11)	0.05391 (9)	0.01593 (19)	
C7	0.24579 (10)	0.47989 (10)	0.16516 (9)	0.01526 (18)	
C8	0.46309 (10)	0.45898 (10)	0.25547 (9)	0.01485 (18)	
C9	0.34030 (10)	0.39846 (10)	0.24473 (8)	0.01406 (18)	
C10	0.33402 (10)	0.27539 (10)	0.19366 (8)	0.01360 (17)	
C11	0.20658 (10)	0.21832 (10)	0.16633 (9)	0.01466 (18)	
C12	0.34409 (10)	0.28881 (11)	0.08242 (8)	0.01450 (18)	
C13	0.19215 (11)	0.24391 (11)	−0.09684 (9)	0.01556 (19)	
C14	0.09432 (11)	0.16901 (12)	−0.15010 (9)	0.0179 (2)	
H14A	0.0581	0.1215	−0.1125	0.022*	
C15	0.05129 (12)	0.16586 (13)	−0.25994 (9)	0.0208 (2)	
H15A	−0.0143	0.1171	−0.2967	0.025*	
C16	0.10863 (12)	0.23713 (13)	−0.31272 (9)	0.0205 (2)	
C17	0.20826 (12)	0.30842 (13)	−0.26164 (9)	0.0212 (2)	
H17A	0.2467	0.3528	−0.2997	0.025*	
C18	0.25023 (12)	0.31284 (13)	−0.15225 (9)	0.0198 (2)	
H18A	0.3164	0.3613	−0.1162	0.024*	
C19	0.28076 (11)	0.47180 (11)	0.38968 (9)	0.01629 (19)	
C20	0.31030 (12)	0.58837 (11)	0.38105 (10)	0.0196 (2)	
H20A	0.3530	0.6100	0.3361	0.024*	
C21	0.27516 (13)	0.67257 (12)	0.44059 (11)	0.0234 (2)	
H21A	0.2947	0.7506	0.4353	0.028*	
C22	0.21111 (13)	0.64049 (14)	0.50775 (10)	0.0250 (3)	
H22A	0.1890	0.6965	0.5481	0.030*	
C23	0.18034 (13)	0.52373 (14)	0.51408 (10)	0.0238 (2)	
H23A	0.1363	0.5024	0.5581	0.029*	
C24	0.21458 (12)	0.43851 (12)	0.45541 (9)	0.0200 (2)	
H24A	0.1937	0.3607	0.4599	0.024*	
C25	0.44075 (10)	0.09116 (10)	0.25076 (9)	0.01556 (19)	
C26	0.47531 (12)	0.02552 (12)	0.34262 (10)	0.0199 (2)	
H26A	0.4875	0.0613	0.4072	0.024*	
C27	0.49152 (14)	−0.09463 (13)	0.33716 (11)	0.0251 (3)	
H27A	0.5149	−0.1390	0.3985	0.030*	
C28	0.47307 (13)	−0.14878 (12)	0.24071 (12)	0.0255 (3)	
H28A	0.4831	−0.2291	0.2373	0.031*	
C29	0.43958 (13)	−0.08168 (12)	0.14961 (11)	0.0242 (2)	
H29A	0.4277	−0.1175	0.0851	0.029*	
C30	0.42349 (13)	0.03901 (12)	0.15369 (10)	0.0208 (2)	
H30A	0.4016	0.0837	0.0926	0.025*	
C31	0.58776 (11)	0.42088 (11)	0.32904 (9)	0.0178 (2)	
H31A	0.6164	0.3561	0.2970	0.021*	
H31B	0.5772	0.3922	0.3931	0.021*	
C32	0.69001 (11)	0.51488 (12)	0.35900 (9)	0.0180 (2)	
H32A	0.6927	0.5522	0.2944	0.022*	
C33	0.66345 (14)	0.60810 (14)	0.42947 (12)	0.0270 (3)	
H33A	0.7272	0.6669	0.4451	0.040*	
H33B	0.6627	0.5729	0.4938	0.040*	
H33C	0.5837	0.6431	0.3938	0.040*	
C34	0.81648 (12)	0.45735 (14)	0.41513 (11)	0.0240 (2)	
H34A	0.8808	0.5156	0.4322	0.036*	
H34B	0.8329	0.3998	0.3697	0.036*	
H34C	0.8150	0.4205	0.4786	0.036*	
C35	0.14649 (11)	0.18458 (11)	0.24507 (9)	0.0181 (2)	
H35A	0.1061	0.2531	0.2611	0.022*	
H35B	0.2115	0.1611	0.3095	0.022*	
C36	0.04988 (12)	0.08607 (12)	0.21154 (10)	0.0205 (2)	
H36A	−0.0092	0.1041	0.1414	0.025*	
C37	0.11105 (18)	−0.03154 (15)	0.20684 (16)	0.0352 (3)	
H37A	0.1548	−0.0271	0.1573	0.053*	
H37B	0.0480	−0.0909	0.1850	0.053*	
H37C	0.1688	−0.0505	0.2753	0.053*	
C38	−0.02151 (14)	0.08170 (16)	0.28949 (12)	0.0294 (3)	
H38A	−0.0841	0.0219	0.2684	0.044*	
H38B	−0.0606	0.1558	0.2902	0.044*	
H38C	0.0355	0.0647	0.3587	0.044*	

### Atomic displacement parameters (Å^2^)

**Table:**

	*U*^11^	*U*^22^	*U*^33^	*U*^12^	*U*^13^	*U*^23^
F1	0.0486 (6)	0.0413 (6)	0.0364 (5)	0.0022 (5)	0.0051 (4)	0.0265 (5)
F2	0.0307 (4)	0.0377 (5)	0.0146 (3)	0.0004 (4)	0.0046 (3)	0.0004 (3)
O1	0.0144 (3)	0.0176 (4)	0.0279 (4)	−0.0001 (3)	0.0071 (3)	0.0017 (4)
O2	0.0164 (3)	0.0223 (4)	0.0205 (4)	−0.0038 (3)	0.0086 (3)	−0.0024 (3)
O3	0.0251 (4)	0.0127 (3)	0.0172 (3)	−0.0003 (3)	0.0110 (3)	−0.0009 (3)
O4	0.0173 (3)	0.0116 (3)	0.0177 (3)	0.0008 (3)	0.0014 (3)	−0.0015 (3)
N1	0.0134 (4)	0.0147 (4)	0.0191 (4)	0.0004 (3)	0.0047 (3)	0.0042 (3)
N2	0.0131 (4)	0.0161 (4)	0.0180 (4)	−0.0004 (3)	0.0035 (3)	0.0008 (4)
N3	0.0153 (4)	0.0175 (4)	0.0171 (4)	−0.0022 (4)	0.0069 (3)	0.0015 (4)
N4	0.0143 (4)	0.0196 (4)	0.0139 (3)	−0.0036 (4)	0.0046 (3)	−0.0001 (3)
C1	0.0208 (5)	0.0258 (6)	0.0217 (5)	0.0003 (5)	0.0092 (4)	0.0035 (5)
C2	0.0284 (6)	0.0332 (7)	0.0229 (5)	−0.0045 (6)	0.0093 (5)	0.0075 (6)
C3	0.0276 (6)	0.0250 (6)	0.0236 (5)	−0.0036 (5)	0.0011 (5)	0.0113 (5)
C4	0.0217 (5)	0.0195 (6)	0.0302 (6)	0.0019 (5)	0.0037 (5)	0.0068 (5)
C5	0.0183 (5)	0.0173 (5)	0.0229 (5)	0.0010 (4)	0.0058 (4)	0.0026 (4)
C6	0.0158 (4)	0.0143 (4)	0.0169 (4)	−0.0010 (4)	0.0045 (3)	0.0023 (4)
C7	0.0156 (4)	0.0125 (4)	0.0185 (4)	0.0004 (4)	0.0068 (3)	0.0006 (4)
C8	0.0146 (4)	0.0128 (4)	0.0166 (4)	−0.0009 (4)	0.0045 (3)	−0.0016 (4)
C9	0.0157 (4)	0.0120 (4)	0.0153 (4)	−0.0003 (4)	0.0063 (3)	−0.0002 (4)
C10	0.0135 (4)	0.0119 (4)	0.0148 (4)	−0.0009 (3)	0.0040 (3)	−0.0011 (4)
C11	0.0156 (4)	0.0126 (4)	0.0163 (4)	−0.0014 (4)	0.0060 (3)	−0.0004 (4)
C12	0.0139 (4)	0.0146 (4)	0.0148 (4)	−0.0002 (4)	0.0046 (3)	−0.0013 (4)
C13	0.0158 (4)	0.0164 (5)	0.0138 (4)	−0.0001 (4)	0.0043 (3)	−0.0002 (4)
C14	0.0176 (5)	0.0182 (5)	0.0174 (4)	−0.0016 (4)	0.0051 (4)	−0.0008 (4)
C15	0.0202 (5)	0.0221 (6)	0.0179 (4)	−0.0014 (5)	0.0035 (4)	−0.0027 (4)
C16	0.0208 (5)	0.0248 (6)	0.0148 (4)	0.0040 (5)	0.0045 (4)	0.0003 (4)
C17	0.0220 (5)	0.0246 (6)	0.0174 (4)	0.0001 (5)	0.0073 (4)	0.0031 (5)
C18	0.0198 (5)	0.0221 (5)	0.0171 (4)	−0.0037 (4)	0.0055 (4)	0.0015 (4)
C19	0.0183 (4)	0.0152 (4)	0.0155 (4)	0.0018 (4)	0.0059 (3)	−0.0017 (4)
C20	0.0221 (5)	0.0153 (5)	0.0225 (5)	0.0000 (4)	0.0088 (4)	−0.0030 (4)
C21	0.0265 (6)	0.0176 (5)	0.0250 (5)	0.0010 (5)	0.0075 (4)	−0.0054 (5)
C22	0.0291 (6)	0.0257 (6)	0.0209 (5)	0.0082 (5)	0.0091 (4)	−0.0044 (5)
C23	0.0275 (6)	0.0270 (6)	0.0201 (5)	0.0088 (5)	0.0119 (4)	0.0032 (5)
C24	0.0235 (5)	0.0200 (5)	0.0190 (4)	0.0031 (4)	0.0101 (4)	0.0021 (4)
C25	0.0135 (4)	0.0122 (4)	0.0195 (4)	0.0007 (4)	0.0037 (3)	−0.0016 (4)
C26	0.0216 (5)	0.0147 (5)	0.0210 (5)	0.0013 (4)	0.0040 (4)	0.0000 (4)
C27	0.0251 (6)	0.0160 (5)	0.0289 (6)	0.0017 (5)	0.0022 (5)	0.0027 (5)
C28	0.0227 (6)	0.0133 (5)	0.0355 (7)	0.0020 (4)	0.0035 (5)	−0.0027 (5)
C29	0.0243 (6)	0.0171 (5)	0.0282 (6)	0.0014 (5)	0.0048 (5)	−0.0078 (5)
C30	0.0242 (5)	0.0158 (5)	0.0204 (5)	0.0018 (4)	0.0052 (4)	−0.0028 (4)
C31	0.0168 (5)	0.0136 (4)	0.0197 (4)	−0.0012 (4)	0.0016 (4)	0.0003 (4)
C32	0.0167 (4)	0.0170 (5)	0.0183 (4)	−0.0022 (4)	0.0032 (4)	−0.0001 (4)
C33	0.0255 (6)	0.0211 (6)	0.0314 (6)	−0.0036 (5)	0.0059 (5)	−0.0087 (5)
C34	0.0171 (5)	0.0246 (6)	0.0263 (5)	−0.0011 (5)	0.0021 (4)	0.0008 (5)
C35	0.0201 (5)	0.0184 (5)	0.0178 (4)	−0.0040 (4)	0.0089 (4)	0.0000 (4)
C36	0.0211 (5)	0.0201 (5)	0.0211 (5)	−0.0054 (5)	0.0080 (4)	0.0020 (4)
C37	0.0394 (8)	0.0184 (6)	0.0518 (9)	−0.0061 (6)	0.0208 (7)	−0.0006 (7)
C38	0.0266 (6)	0.0362 (8)	0.0286 (6)	−0.0095 (6)	0.0135 (5)	0.0045 (6)

### Geometric parameters (Å, °)

**Table:**

F1—C3	1.3570 (17)	C20—C21	1.3975 (18)
F2—C16	1.3620 (14)	C20—H20A	0.9300
O1—C7	1.2136 (14)	C21—C22	1.391 (2)
O2—C12	1.2169 (14)	C21—H21A	0.9300
O3—C19	1.3880 (15)	C22—C23	1.393 (2)
O3—C9	1.4138 (13)	C22—H22A	0.9300
O4—C25	1.3981 (15)	C23—C24	1.3930 (19)
O4—C10	1.4166 (14)	C23—H23A	0.9300
N1—C7	1.3693 (15)	C24—H24A	0.9300
N1—N2	1.4179 (14)	C25—C26	1.3879 (17)
N1—C6	1.4200 (15)	C25—C30	1.3914 (16)
N2—C8	1.2901 (15)	C26—C27	1.3943 (19)
N3—C11	1.2843 (14)	C26—H26A	0.9300
N3—N4	1.4131 (13)	C27—C28	1.392 (2)
N4—C12	1.3680 (15)	C27—H27A	0.9300
N4—C13	1.4180 (14)	C28—C29	1.389 (2)
C1—C2	1.392 (2)	C28—H28A	0.9300
C1—C6	1.3949 (17)	C29—C30	1.3985 (19)
C1—H1A	0.9300	C29—H29A	0.9300
C2—C3	1.384 (2)	C30—H30A	0.9300
C2—H2A	0.9300	C31—C32	1.5367 (17)
C3—C4	1.380 (2)	C31—H31A	0.9700
C4—C5	1.3977 (18)	C31—H31B	0.9700
C4—H4A	0.9300	C32—C33	1.5265 (19)
C5—C6	1.3910 (17)	C32—C34	1.5313 (18)
C5—H5A	0.9300	C32—H32A	0.9800
C7—C9	1.5492 (16)	C33—H33A	0.9600
C8—C31	1.4961 (16)	C33—H33B	0.9600
C8—C9	1.5232 (16)	C33—H33C	0.9600
C9—C10	1.5604 (16)	C34—H34A	0.9600
C10—C11	1.5192 (16)	C34—H34B	0.9600
C10—C12	1.5506 (15)	C34—H34C	0.9600
C11—C35	1.4956 (15)	C35—C36	1.5338 (18)
C13—C14	1.3974 (17)	C35—H35A	0.9700
C13—C18	1.3978 (17)	C35—H35B	0.9700
C14—C15	1.3949 (16)	C36—C37	1.528 (2)
C14—H14A	0.9300	C36—C38	1.5320 (18)
C15—C16	1.3836 (19)	C36—H36A	0.9800
C15—H15A	0.9300	C37—H37A	0.9600
C16—C17	1.3787 (19)	C37—H37B	0.9600
C17—C18	1.3898 (16)	C37—H37C	0.9600
C17—H17A	0.9300	C38—H38A	0.9600
C18—H18A	0.9300	C38—H38B	0.9600
C19—C20	1.3918 (18)	C38—H38C	0.9600
C19—C24	1.3969 (16)		
			
C19—O3—C9	119.95 (10)	C22—C21—H21A	119.8
C25—O4—C10	119.19 (9)	C20—C21—H21A	119.8
C7—N1—N2	113.63 (9)	C21—C22—C23	119.47 (12)
C7—N1—C6	127.08 (10)	C21—C22—H22A	120.3
N2—N1—C6	119.28 (9)	C23—C22—H22A	120.3
C8—N2—N1	108.28 (10)	C22—C23—C24	120.93 (12)
C11—N3—N4	108.68 (9)	C22—C23—H23A	119.5
C12—N4—N3	113.12 (9)	C24—C23—H23A	119.5
C12—N4—C13	128.13 (10)	C23—C24—C19	118.93 (13)
N3—N4—C13	118.52 (9)	C23—C24—H24A	120.5
C2—C1—C6	119.15 (13)	C19—C24—H24A	120.5
C2—C1—H1A	120.4	C26—C25—C30	120.97 (11)
C6—C1—H1A	120.4	C26—C25—O4	114.99 (10)
C3—C2—C1	118.66 (12)	C30—C25—O4	123.91 (11)
C3—C2—H2A	120.7	C25—C26—C27	119.38 (12)
C1—C2—H2A	120.7	C25—C26—H26A	120.3
F1—C3—C4	118.35 (15)	C27—C26—H26A	120.3
F1—C3—C2	118.36 (14)	C28—C27—C26	120.58 (13)
C4—C3—C2	123.29 (13)	C28—C27—H27A	119.7
C3—C4—C5	117.83 (13)	C26—C27—H27A	119.7
C3—C4—H4A	121.1	C29—C28—C27	119.32 (13)
C5—C4—H4A	121.1	C29—C28—H28A	120.3
C6—C5—C4	119.90 (12)	C27—C28—H28A	120.3
C6—C5—H5A	120.0	C28—C29—C30	120.86 (13)
C4—C5—H5A	120.0	C28—C29—H29A	119.6
C5—C6—C1	121.16 (11)	C30—C29—H29A	119.6
C5—C6—N1	120.23 (10)	C25—C30—C29	118.88 (13)
C1—C6—N1	118.61 (11)	C25—C30—H30A	120.6
O1—C7—N1	128.33 (11)	C29—C30—H30A	120.6
O1—C7—C9	127.19 (11)	C8—C31—C32	115.77 (10)
N1—C7—C9	104.43 (9)	C8—C31—H31A	108.3
N2—C8—C31	123.48 (11)	C32—C31—H31A	108.3
N2—C8—C9	112.06 (10)	C8—C31—H31B	108.3
C31—C8—C9	124.40 (10)	C32—C31—H31B	108.3
O3—C9—C8	116.03 (9)	H31A—C31—H31B	107.4
O3—C9—C7	114.68 (9)	C33—C32—C34	110.15 (11)
C8—C9—C7	101.08 (9)	C33—C32—C31	111.23 (10)
O3—C9—C10	105.36 (9)	C34—C32—C31	109.31 (11)
C8—C9—C10	110.92 (9)	C33—C32—H32A	108.7
C7—C9—C10	108.72 (9)	C34—C32—H32A	108.7
O4—C10—C11	114.78 (9)	C31—C32—H32A	108.7
O4—C10—C12	114.02 (9)	C32—C33—H33A	109.5
C11—C10—C12	100.65 (9)	C32—C33—H33B	109.5
O4—C10—C9	105.08 (8)	H33A—C33—H33B	109.5
C11—C10—C9	113.18 (9)	C32—C33—H33C	109.5
C12—C10—C9	109.28 (9)	H33A—C33—H33C	109.5
N3—C11—C35	122.94 (10)	H33B—C33—H33C	109.5
N3—C11—C10	112.42 (9)	C32—C34—H34A	109.5
C35—C11—C10	124.59 (10)	C32—C34—H34B	109.5
O2—C12—N4	128.57 (10)	H34A—C34—H34B	109.5
O2—C12—C10	126.50 (10)	C32—C34—H34C	109.5
N4—C12—C10	104.92 (9)	H34A—C34—H34C	109.5
C14—C13—C18	120.75 (10)	H34B—C34—H34C	109.5
C14—C13—N4	119.55 (10)	C11—C35—C36	115.82 (10)
C18—C13—N4	119.70 (10)	C11—C35—H35A	108.3
C15—C14—C13	119.65 (11)	C36—C35—H35A	108.3
C15—C14—H14A	120.2	C11—C35—H35B	108.3
C13—C14—H14A	120.2	C36—C35—H35B	108.3
C16—C15—C14	118.28 (12)	H35A—C35—H35B	107.4
C16—C15—H15A	120.9	C37—C36—C38	110.78 (13)
C14—C15—H15A	120.9	C37—C36—C35	111.69 (11)
F2—C16—C17	118.07 (12)	C38—C36—C35	108.41 (11)
F2—C16—C15	118.95 (12)	C37—C36—H36A	108.6
C17—C16—C15	122.98 (11)	C38—C36—H36A	108.6
C16—C17—C18	118.80 (12)	C35—C36—H36A	108.6
C16—C17—H17A	120.6	C36—C37—H37A	109.5
C18—C17—H17A	120.6	C36—C37—H37B	109.5
C17—C18—C13	119.47 (12)	H37A—C37—H37B	109.5
C17—C18—H18A	120.3	C36—C37—H37C	109.5
C13—C18—H18A	120.3	H37A—C37—H37C	109.5
O3—C19—C20	125.06 (10)	H37B—C37—H37C	109.5
O3—C19—C24	114.06 (11)	C36—C38—H38A	109.5
C20—C19—C24	120.84 (11)	C36—C38—H38B	109.5
C19—C20—C21	119.35 (12)	H38A—C38—H38B	109.5
C19—C20—H20A	120.3	C36—C38—H38C	109.5
C21—C20—H20A	120.3	H38A—C38—H38C	109.5
C22—C21—C20	120.46 (13)	H38B—C38—H38C	109.5
			
C7—N1—N2—C8	2.00 (14)	O4—C10—C11—C35	57.29 (15)
C6—N1—N2—C8	−176.81 (11)	C12—C10—C11—C35	−179.82 (11)
C11—N3—N4—C12	3.32 (15)	C9—C10—C11—C35	−63.33 (15)
C11—N3—N4—C13	178.24 (11)	N3—N4—C12—O2	174.88 (13)
C6—C1—C2—C3	−0.8 (2)	C13—N4—C12—O2	0.6 (2)
C1—C2—C3—F1	−179.36 (14)	N3—N4—C12—C10	−4.77 (13)
C1—C2—C3—C4	0.9 (2)	C13—N4—C12—C10	−179.10 (11)
F1—C3—C4—C5	179.90 (13)	O4—C10—C12—O2	−52.06 (16)
C2—C3—C4—C5	−0.4 (2)	C11—C10—C12—O2	−175.47 (12)
C3—C4—C5—C6	−0.3 (2)	C9—C10—C12—O2	65.18 (15)
C4—C5—C6—C1	0.4 (2)	O4—C10—C12—N4	127.61 (10)
C4—C5—C6—N1	−179.47 (12)	C11—C10—C12—N4	4.19 (12)
C2—C1—C6—C5	0.1 (2)	C9—C10—C12—N4	−115.16 (10)
C2—C1—C6—N1	−179.99 (12)	C12—N4—C13—C14	158.41 (13)
C7—N1—C6—C5	49.72 (18)	N3—N4—C13—C14	−15.66 (17)
N2—N1—C6—C5	−131.64 (12)	C12—N4—C13—C18	−22.5 (2)
C7—N1—C6—C1	−130.14 (13)	N3—N4—C13—C18	163.43 (12)
N2—N1—C6—C1	48.49 (16)	C18—C13—C14—C15	−2.19 (19)
N2—N1—C7—O1	176.51 (12)	N4—C13—C14—C15	176.88 (12)
C6—N1—C7—O1	−4.8 (2)	C13—C14—C15—C16	0.63 (19)
N2—N1—C7—C9	−5.82 (13)	C14—C15—C16—F2	−178.52 (12)
C6—N1—C7—C9	172.88 (11)	C14—C15—C16—C17	1.8 (2)
N1—N2—C8—C31	−179.34 (10)	F2—C16—C17—C18	177.67 (12)
N1—N2—C8—C9	3.06 (13)	C15—C16—C17—C18	−2.7 (2)
C19—O3—C9—C8	74.67 (13)	C16—C17—C18—C13	1.0 (2)
C19—O3—C9—C7	−42.71 (14)	C14—C13—C18—C17	1.3 (2)
C19—O3—C9—C10	−162.22 (10)	N4—C13—C18—C17	−177.73 (12)
N2—C8—C9—O3	−130.90 (11)	C9—O3—C19—C20	−25.83 (17)
C31—C8—C9—O3	51.53 (15)	C9—O3—C19—C24	156.49 (11)
N2—C8—C9—C7	−6.20 (12)	O3—C19—C20—C21	−176.32 (11)
C31—C8—C9—C7	176.23 (10)	C24—C19—C20—C21	1.21 (19)
N2—C8—C9—C10	108.96 (11)	C19—C20—C21—C22	−0.1 (2)
C31—C8—C9—C10	−68.62 (13)	C20—C21—C22—C23	−1.0 (2)
O1—C7—C9—O3	−49.90 (16)	C21—C22—C23—C24	1.0 (2)
N1—C7—C9—O3	132.39 (10)	C22—C23—C24—C19	0.0 (2)
O1—C7—C9—C8	−175.51 (12)	O3—C19—C24—C23	176.61 (11)
N1—C7—C9—C8	6.79 (11)	C20—C19—C24—C23	−1.18 (18)
O1—C7—C9—C10	67.71 (15)	C10—O4—C25—C26	−139.56 (11)
N1—C7—C9—C10	−109.99 (10)	C10—O4—C25—C30	44.49 (16)
C25—O4—C10—C11	44.64 (14)	C30—C25—C26—C27	−0.73 (19)
C25—O4—C10—C12	−70.74 (13)	O4—C25—C26—C27	−176.81 (12)
C25—O4—C10—C9	169.63 (9)	C25—C26—C27—C28	−0.2 (2)
O3—C9—C10—O4	−63.88 (10)	C26—C27—C28—C29	0.7 (2)
C8—C9—C10—O4	62.43 (11)	C27—C28—C29—C30	−0.4 (2)
C7—C9—C10—O4	172.73 (9)	C26—C25—C30—C29	1.0 (2)
O3—C9—C10—C11	62.11 (11)	O4—C25—C30—C29	176.74 (12)
C8—C9—C10—C11	−171.58 (9)	C28—C29—C30—C25	−0.4 (2)
C7—C9—C10—C11	−61.28 (11)	N2—C8—C31—C32	24.85 (17)
O3—C9—C10—C12	173.38 (9)	C9—C8—C31—C32	−157.85 (11)
C8—C9—C10—C12	−60.31 (11)	C8—C31—C32—C33	69.65 (14)
C7—C9—C10—C12	49.99 (11)	C8—C31—C32—C34	−168.49 (10)
N4—N3—C11—C35	177.20 (11)	N3—C11—C35—C36	27.87 (18)
N4—N3—C11—C10	−0.15 (14)	C10—C11—C35—C36	−155.11 (11)
O4—C10—C11—N3	−125.42 (11)	C11—C35—C36—C37	69.94 (15)
C12—C10—C11—N3	−2.52 (13)	C11—C35—C36—C38	−167.73 (12)
C9—C10—C11—N3	113.96 (11)		

### Hydrogen-bond geometry (Å, °)

**Table:**

*Cg1* is the centroid of the N2═C8 double bond. *Cg3* and *Cg5* are the centroids of the C1–C6 and C19–C24 rings, respectively.

**Table:**

*D*—H···*A*	*D*—H	H···*A*	*D*···*A*	*D*—H···*A*
C15—H15A···Cg5^i^	0.93	2.82	3.6941 (14)	157
C20—H20A···Cg1	0.93	2.54	2.9632 (12)	135
C29—H29A···Cg3^ii^	0.93	2.82	3.4701 (15)	128
C36—H36A···Cg3^i^	0.98	2.91	3.7529 (15)	145

Symmetry codes: (i) −*x*, *y*−1/2, −*z*; (ii) *x*, *y*−1, *z*.
